# Sacituzumab Govitecan for the treatment of advanced triple negative breast cancer patients: a multi-center real-world analysis

**DOI:** 10.3389/fonc.2024.1362641

**Published:** 2024-03-26

**Authors:** Roberta Caputo, Giuseppe Buono, Michela Piezzo, Claudia Martinelli, Daniela Cianniello, Alessandro Rizzo, Francesco Pantano, Nicoletta Staropoli, Rodolfo Cangiano, Salvatore Turano, Ida Paris, Francesco Nuzzo, Alessandra Fabi, Michelino De Laurentiis

**Affiliations:** ^1^ Department of Breast and Thoracic Oncology, Istituto Nazionale Tumori – IRCCS- “Fondazione G. Pascale", Naples, Italy; ^2^ Department of Clinical Medicine and Surgery, University of Naples Federico II, Naples, Italy; ^3^ Clinical and Translational Oncology, Scuola Superiore Meridionale (SSM), Naples, Italy; ^4^ Medical Oncology Department, I.R.C.C.S. Istituto Tumori ”Giovanni Paolo II”, Bari, Italy; ^5^ Department of Medical Oncology, Fondazione Policlinico Universitario Campus Bio-Medico, Rome, Italy; ^6^ Medical Oncology and Translational Medical Oncology Units, Department of Experimental and Clinical Medicine, Magna Graecia University, AOU R. Dulbecco Catanzaro, Catanzaro, Italy; ^7^ UOSD Oncologia PO Piedimonte Matese, ASL Caserta, Caserta, Italy; ^8^ Department of Oncohematology, UO Oncologia Azienda Ospedaliera di Cosenza, Cosenza, Italy; ^9^ Gynecologic Oncology, Department of Woman and Child Health and Public Health, Woman Health Area, Fondazione Policlinico Universitario A. Gemelli IRCCS, Rome, Italy; ^10^ Precision Medicine in Senology, Scientific Directorate – Department of Women and Child Health, Fondazione Policlinico Universitario A. Gemelli IRCCS, Rome, Italy

**Keywords:** Sacituzumab govitecan, triple negative, metastatic breast cancer, retrospective study, Italy

## Abstract

**Objective:**

The objective of this multicenter, observational, retrospective analysis was to evaluate the safety and efficacy of sacituzumab govitecan in metastatic triple-negative breast cancer (mTNBC) patients managed according to common clinical practice in Italy.

**Methods:**

Data were retrieved by 7 sites. Triple-negative BC was defined by the lack of expression of estrogen receptor (ER <1%), progesterone receptor (PgR <1%) and human-epidermal growth factor receptor-2 (HER2 0, 1+, 2+ ISH-not amplified) according to standard ASCO-CAP criteria. Demographic and clinical characteristics were collected. Premedication, dose modifications and treatment schedule were based on the approved label of the product. Adverse events (AEs) were assessed according to NCI-CTCAE v5.0.

**Results:**

Fifty-seven eligible patients who received sacituzumab govitecan for mTNBC were included. Median age was 53 years (range 25-75). Approximately 70% of patients had an initial diagnosis of TNBC. Median time from the diagnosis of metastatic BC to start of sacituzumab govitecan was 17 months (range 0-97) and median number of previous therapies was 3 (range 1-7). The most common sites of metastasis were lymph nodes (63.1% of patients), lung (57.9%), bone (50.8%) and liver (38.6%). Eight (14.0%) patients had a disease-free interval ≤12 months. A total of 32 (56.1%) deaths were observed and the median overall survival (OS) was 12.43 months (95% CI, 7.97 months-not reached). At a median follow-up of 10.6 months, 45 patients (78.9%) had progression and the median progression-free survival (PFS) was 4.9 months (95% CI, 3.7-7.1 months). Partial tumour response was observed in 19 patients (33.3%), stable disease in 16 (28.1%) and disease progression in 22 patients (38.6%). The most common treatment-related AEs were anemia (66.6% of patients), alopecia (66.6%), neutropenia (59.6%), nausea (42.1%) and diarrhea (38.6%). Neutropenia was the most common serious treatment-related AE: 21.0% and 8.7% of patients experienced grade 3 or 4 neutropenia, respectively. Twenty-two patients (38.6%) reduced the dose and 5.3% permanently discontinued treatment.

**Conclusion:**

The results of this real-world analysis showed that both safety and efficacy of sacituzumab govitecan in mTNBC patients are consistent with that previously reported in regulatory trials. The use of premedication and supportive measures was associated with a satisfactory toxicity profile.

## 1 Introduction

Triple negative breast cancer (TNBC), defined by a lack of tumor-cell expression of the estrogen receptor (ER), progesterone receptor (PR), and human epidermal growth factor receptor 2 (HER2), accounts for approximately 15-20% of all BCs (1). This subgroup is characterized by a worse prognosis and a poorer survival outcome compared to hormone receptor (HR)-positive and HER2-positive BCs, despite high chemo-sensitivity, which represents the so-called TNBC paradox (1). In fact, is estimated that more than half of treated TNBC patients with localized disease are likely to experience disease relapse within the first 5 years from diagnosis (2, 3).

Currently, there are limited treatment options for the management of metastatic TNBC (mTNBC), with cytotoxic chemotherapy still having a predominant role, acting as the backbone of treatment (4, 5). However, in the metastatic setting, chemotherapy is commonly associated with low tumor response and early disease progression, with an estimated median progression-free survival (PFS) and overall survival (OS) of only 2-3 and 10.2 months, respectively (2).

In the aim to improve mTNBC patients’ outcome, a wide range of novel therapies are currently under investigation, with some of them also recently approved. Among these, antibody-drug conjugates (ADCs) represent a new class of drug designed to specifically deliver high-potent chemotherapeutic agents directly to cancer cells, using the affinity between the antibody and the target antigen, which is hyper-expressed on cancer cell (6).

Sacituzumab govitecan represents the first ADC approved by the Food and Drug Administration (FDA) and the European Medicines Agency (EMA) for the treatment of unresectable locally-advanced or mTNBC patients who have received two or more prior systemic therapies, at least one of them for metastatic disease (7). It consists of three main parts: 1) a humanized monoclonal antibody (hRS7), which selectively binds human trophoblastic cell surface antigen 2 (Trop-2), a transmembrane glycoprotein that is highly expressed in many solid tumors, including TNBC (8, 9); 2) an hydrolysable linker (CL2A), which facilitates rapid internalization and efficient release of the payload in Trop-2-expressing cancer cells and into the surrounding tumor microenvironment; 3) the payload, SN-38, an active metabolite of the irinotecan, which interacts with topoisomerase I and prevents re-ligation of topoisomerase I-induced single strand breaks; this results in DNA damage, ultimately leading to apoptosis and cell death (7).

This new ADC received FDA accelerated approval in April 2020, based on the results of the phase 1-2 trial IMMU-132-01, where the treatment with sacituzumab govitecan, in a cohort of 108 patients with mTNBC, was associated with an objective response rate (ORR) of 33%, a median PFS of 5.5 months and a median OS of 13.0 months (10). Subsequently, the confirmatory phase 3 ASCENT trial showed that in 468 patients with relapsed or refractory mTNBC, sacituzumab govitecan was associated with a significant benefit over chemotherapy of physician’s choice (eribulin, vinorelbine, capecitabine, or gemcitabine) in terms of ORR (35% vs 5%), median PFS (5.6 vs 1.7 months) and median OS (12.1 vs 6.7 months) (11). Myelosuppression and diarrhea were the most common toxicities reported in patients treated with sacituzumab govitecan (11). Subgroup analyses of the ASCENT trial demonstrated treatment benefit for the experimental drug irrespective of the presence of several baseline clinical features, including initial TNBC diagnosis (12), brain metastases (13), age, number of prior therapies, and prior therapy with immune checkpoint inhibitors (11). Finally, a better quality of life was also observed in the sacituzumab govitecan treated patients compared to chemotherapy group (14).

Recently, sacituzumab govitecan has been also approved in several countries for the treatment of metastatic endocrine-resistant HR-positive/HER2-negative BC, based on the results of the phase III TROPiCS-02 trial (15).

There are very few published reports describing the efficacy and safety of sacituzumab govitecan in mTNBC in a real-world setting. To our knowledge, at the time of the current analysis, only one observational single-center study conducted in Germany on 43 patients was published (16), showing an efficacy and safety profile similar to that observed in regulatory trials.

Considering this background and taking into account that no Italian centers participated in the ASCENT trial, we considered of interest to conduct a multicenter, observational, retrospective analysis on the safety and efficacy of sacituzumab govitecan in a cohort of mTNBC patients managed according to common clinical practice in Italy.

## 2 Materials and methods

### 2.1 Patients

The study population represents a retrospective cohort of women enrolled in the study NCT02284581, a retrospective and prospective observational multicenter study, aimed to identify the duration of treatments (chemotherapy, hormonal therapy and biological therapies) according to biological subtype and line of treatment in mTNBC patients. Patients included in the present analysis were selected based on the following criteria:

- patients ≥ 18 years of age;
- histologically confirmed diagnosis of mTNBC (*de novo* or relapsed disease);
- treatment with sacituzumab govitecan following at least two previous standard chemotherapy regimens;
- availability of efficacy and safety data needed for the purpose of the analysis;
- patients who received sacituzumab govitecan within a randomized clinical trial were excluded.

Triple negative BC was defined according to standard American Society of Clinical Oncology-College of American Pathologists criteria (17), i.e. ER<1%, PgR<1%, HER2:0 or 1+,2+, FISH: not amplified. Metastatic disease was documented per RECIST criteria, version 1.1 (18).

### 2.2 Investigational product

According to the EMA approved summary of product characteristic (SmPC) (7), sacituzumab govitecan was administered as an intravenous infusion at the dose of 10 mg/kg body once weekly on Day 1 and Day 8 of 21-day treatment cycles, to be continued until disease progression or unacceptable toxicity. Dose modification in case of infusion-related reactions or adverse reactions was according to the approved SmPC of the product.

### 2.3 Study endpoints

The efficacy endpoints of the study were: PFS, defined as the time elapsing between the date of first administration of sacituzumab govitecan and the date of the first evidence of progression or death, whichever occurred first; OS, defined as the time elapsing between the date of first administration of sacituzumab govitecan and the date of death due to any cause; and objective tumor response (RECIST 1.1 criteria). Data of patients not in progression at the last follow-up were considered as censored for PFS, and data of patients alive at the last follow-up were considered as censored for both OS and PFS. Safety was evaluated by recording of adverse events (AEs) related to sacituzumab govitecan, which were assessed according to National Cancer Institute Common Terminology Criteria for Adverse Events (NCI-CTCAE), version 5.0.

### 2.4 Statistics

Sample characteristics were presented using descriptive statistics, i.e. median with range for numerical variables, and absolute frequencies and percentages for categorical variables. PFS and OS (and median follow-up for the two endpoints) were analyzed using the reverse Kaplan-Meier estimates; median values and 95% confidence interval (CI) were presented. Treatment-related AEs terms were assigned to a Preferred Term (PT) and were classified by the primary System Organ Class (SOC) according to the Medical Dictionary for Regulatory Activities (MedDRA) thesaurus, version 23.0. The worst (i.e. the higher) NCI-CTCAE toxicity grade was used for the treatment-related AEs reported more than once in the same patient.

Data analysis included all patients who had started treatment with sacituzumab govitecan. All statistical analyses were conducted using the statistical platform R (version 3.6.1).

### 2.5 Ethics

The study was approved by the institutional review boards and a written informed consent was required for each participant.

## 3 Results

### 3.1 Characteristics of patients

Demographics and clinical characteristics are described in **Table 1**. The overall cohort included 57 patients enrolled in 7 sites in Italy, who started treatment with sacituzumab govitecan between March 2021 and March 2023 with a median follow up of 10.6 months. The median age at time of treatment initiation was 53 years (range 25-75 years). Approximately 70% of patients had a diagnosis of triple negative BC at initial diagnosis. The median time from the diagnosis of mTNBC and start of treatment with sacituzumab govitecan was 17 months (range 0-97 months). The most common sites of metastasis were the lymph nodes (36 patients, 63.1%), the lung (33 patients, 57.9%) and the bone (29 patients, 50.8%). Liver metastases were reported in approximately 40% of patients. Approximately half of patients had ≥3 metastatic sites and 14% of patients had only one metastatic site.

**Table 1:** **S**ummary of demographics and baseline characteristics of patients.

Characteristics	N = 57
Age, year	
Median (range)	53 (25-75)
ECOG performance-status, N (%)	
0-1	55 (96.5%)
2	2 (3.5%)
Germline BRCA1 or BRCA2 mutation status, N (%)	
Negative	28 (49.1%)
Positive	8 (14.0%)
Unknown	21 (36.9%)
Triple-negative breast cancer at initial diagnosis, N (%)	
Yes	40 (70.2%)
No	17 (29.8%)
HER2-low breast cancer at initial diagnosis, N (%)	
Yes	17 (29.8%)
No	40 (70.2%)
ER-low breast cancer at initial diagnosis, N (%)	
Yes	4 (7.0%)
No	53 (93.0%)
Disease Free Interval, N (%)	
*De novo*	5 (8.8%)
≤ 12 months	8 (14.0%)
≥24 months	44 (77.2%)
Time from diagnosis of metastatic disease to sacituzumab, months govitecaninitiation, months	
Median (range)	17 (0-97)
Major tumor locations, N (%)	
Lung	33 (57.9%)
Liver	22 (38.6%)
Lymph nodes	36 (63.1%)
Bone*	29 (50.8%)
Brain	16 (28.1%)
Number of metastatic sites, N (%)	
1	8 (14.0%)
2	22 (38.6%)
≥3	27 (47.4%)
Number of previous anticancer regimens	
Median (range)	3 (1-7)
Previous use of PARP immune checkpoint inhibitors, N (%)	
Yes	4 (7.1%)
No	53 (92.9%)
Previous use of PD-1 or PD-L1 PARP immune checkpoint inhibitors inhibitors, N(%)	
Yes	15 (26.3%)
No	42 (73.7%)

Patients received a median of 3 (range 1-7) previous anticancer regimens for metastatic disease. Four (7.1%) and 15 (26.3%) patients were previously treated with PARP inhibitors and/or prior PD-1 or PD-L1 immune checkpoint inhibitors, respectively. Eight patients (14%) had a disease-free interval ≤ 12 months.

**Table 2** shows the premedications used. All patients received a premedication 30 minutes before starting the infusion. The most common were corticosteroids (52 patients, 91.2%), serotonin (5-HT 3)-receptor and neurokinin type 1 (NK 1)-receptor antagonists at baseline (before 1^st^ cycle) (43 patients, 75.4%) for the prevention of chemotherapy-induced nausea and vomiting (CINV). Interestingly, 15 patients (26.3%) used prophylactic atropine to prevent cholinergic syndrome, while only 4 patients (7%) required it to treat this syndrome. Prophylactic atropine was used in cases of acute diarrhea (grade ≤2) during or shortly after the infusion and in cases of cholinergic syndrome. It was also used from the first cycle in patients at high risk of developing acute diarrhea or cholinergic syndrome. Delayed diarrhea (grade ≤2) was more commonly reported after day 8 of the first cycle and was managed with loperamide (up to 16 mg/day).

**Table 2:** Premedications and preventive treatments used in the study .

Used premedication	N = 57
Corticosteroids, N (%)	52 (91.2%)
5-HT 3 receptor antagonist alone, N (%)	18 (31.6%)
5-HT 3 + NK 1 receptor antagonists at baseline (before 1^st^ cycle), N (%)	43 (75.4%)
5-HT 3 + NK 1 receptor antagonists because of side effects, N (%)	6 (10.5%)
Atropine at baseline (before 1^st^ cycle), N (%)	15 (26.3%)
Atropine because of side effects, N (%)	4 (7.0%)

a patient may have received more than one treatment.

Data are number (N) and percentage (%) of patients.

### 3.2 Safety results

**Table 3** shows the summary of treatment-related AEs. Overall, 55 patients (96.5%) reported any-grade treatment-related AEs, while 22 (38.6%) and 6 (10.5%) patients reported grade 3 and grade 4 AEs, respectively. Anemia (38 patients, 66.6%), alopecia (38 patients, 66.6%), neutropenia (34 patients, 59.6%), nausea (24 patients, 42.1%) and diarrhea (22 patients, 38.6%) were the most common treatment-related AEs (any grade). Overall, 28 patients (49.1%) experienced serious AEs (grade 3: 22 patients, 38.6%; grade 4: 6 patients, 10.5%). Among these, neutropenia represented the most common one (grade 3: 12 patients, 21.0%; grade 4: 5 patients, 8.7%), followed by ALT/AST increased (grade 3: 3 patients, 5.3%; grade 4: 1 patient, 1.7%). Apart from 2 cases (3.5%) of grade 3 diarrhea, none of the other grade 3-4 treatment-related AEs were reported in more than one patient.

**Table 3:** Summary of treatment-related adverse events by system organ class and preferred term, overall and by grade (3 or 4).

System organ class Preferred term	N = 57		
	Any grade	Grade 3	Grade 4
Any adverse event	55 (96.4%)	22 (38.6%)	6 (10.5)
Blood and lymphatic system disorders			
Neutropenia, N (%)	34 (59.6%)	12 (21.0%)	5 (8.7%)
Anemia, N (%)	38 (66.6%)	1 (1.7%)	0
Thrombocytopenia, N (%)	15 (26.3%)	0	0
Gastrointestinal disorders			
Nausea, N (%)	24 (42.1%)	0	0
Diarrhea, N (%)	24 (42.1%)	2 (3.5%)	0
Constipation, N (%)	3 (5.2%)	0	0
Vomiting, N (%)	5 (8.7%)	0	0
Abdominal pain, N (%)	5 (8.7%)	0	0
Gastralgia, N (%)	1 (1.7%)	1 (1.7%)	0
Erosive gastritis, N (%)	1 (1.7%)	1 (1.7%)	0
General disorders and administration-site conditions			
Asthenia, N (%)	12 (21.0%)	1 (1.7%)	0
Fatigue, N (%)	5 (8.7%)	0	0
Skin and subcutaneous disorders			
Pruritus, N (%)	2 (3.5%)	0	0
Alopecia, N (%)	38 (66.6%)	0	0
Skin rash, N (%)	1 (1.7%)	0	0
Skin hyperchromia, N (%)	1 (1.7%)	0	0
Metabolism and nutrition disorders			
Hyperglycemia, N (%)	3 (5.2%)	0	0
Nervous system disorders and psychiatric disorders			
Dysgeusia, N (%)	2 (3.5%)	0	0
Dizziness, N (%)	1 (1.7%)	0	0
Insomnia, N (%)	1 (1.7%)	0	0
Investigations			
ALT/AST increased, N (%)	14 (24.6%)	3 (5.3%)	1 (1.7%)
Amylase/Lipase increased, N (%)	1 (1.7%)	1 (1.7%)	0
Bilirubin increased, N (%)	2 (3.5%)	0	0

Data are number (N) and percentage (%) of patients.

Three patients (5.3%) permanently discontinued treatment and 22 patients (38.6%) reduced the dose of sacituzumab-govitecan due to toxicity. No hypersensitivity reactions occurred.

### 3.3 Efficacy results

**Figure 1:**
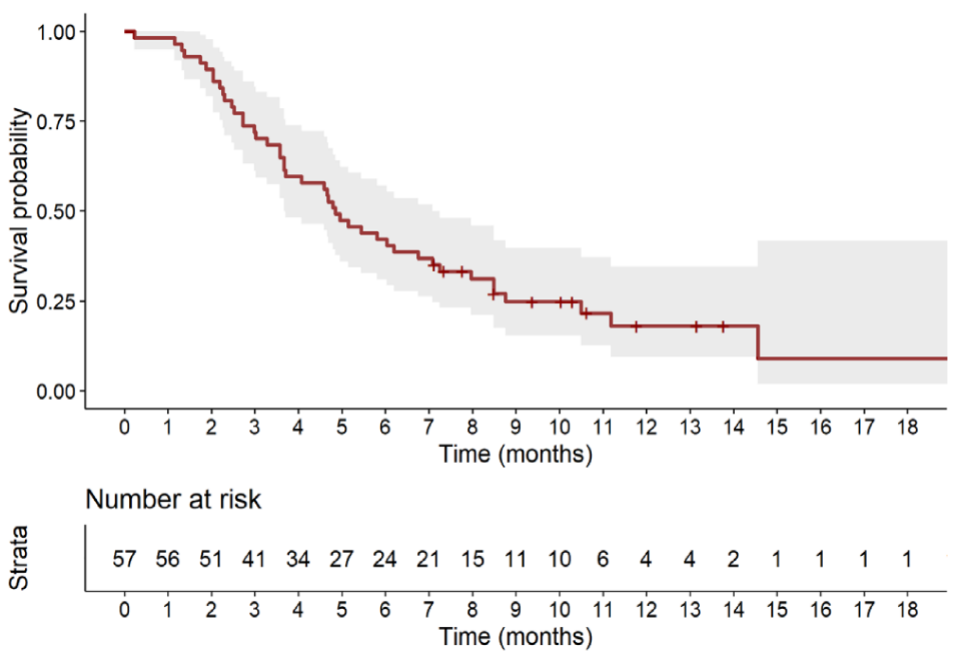
Kaplan-Meier estimate of progression-free survival up to 18 months. Full line shows the median value and shaded space shows the 95% CI.

**Figure 1** shows the Kaplan-Meier estimate of PFS up to 18 months. At a median follow-up of 10.6 months (95% CI, 10.0 months to not evaluable), 45 patients (78.9%) had tumor progression or died. The median PFS was 4.9 months (95% CI, 3.7 to 7.1 months).

**Figure 2:**
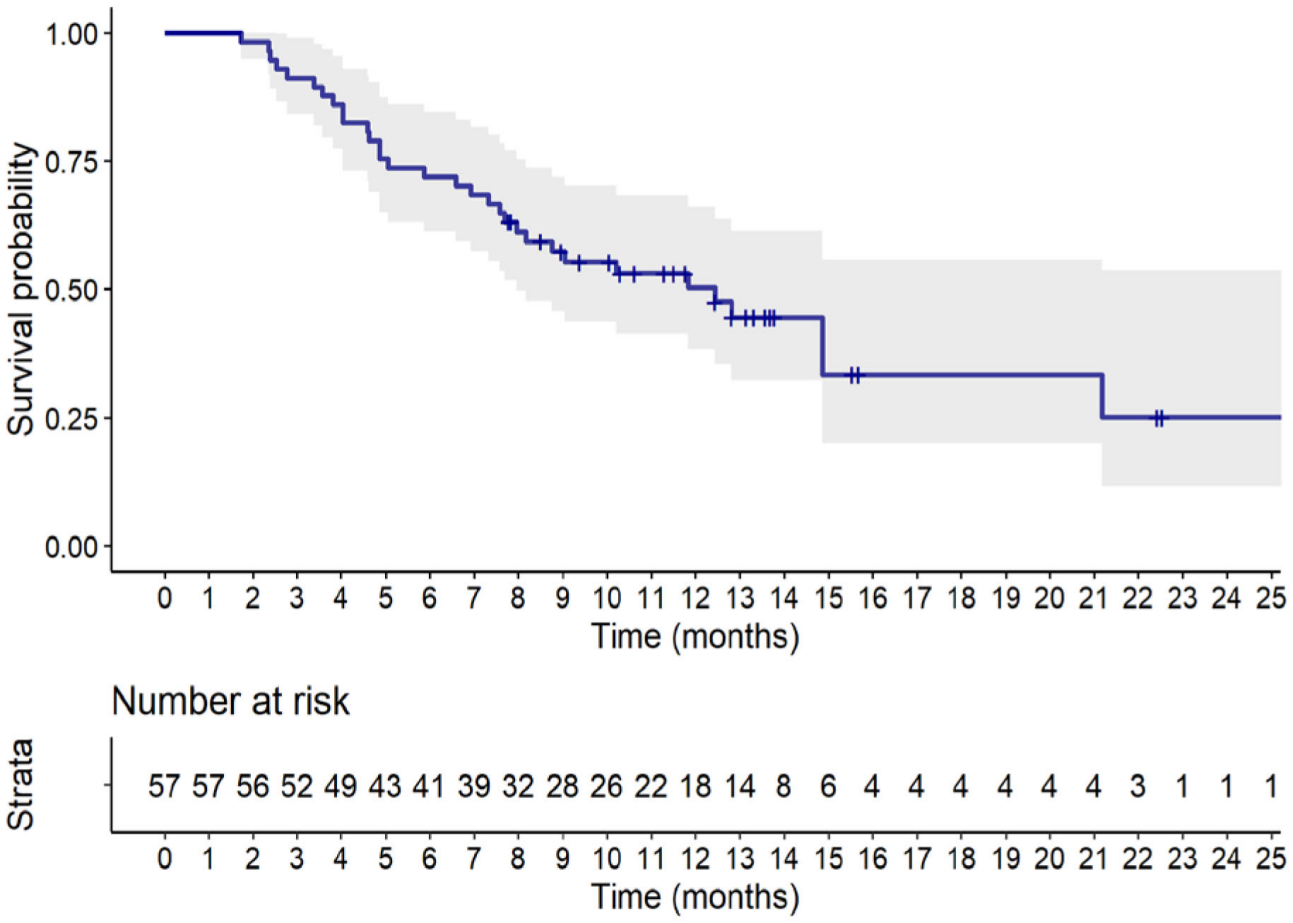
Kaplan-Meier estimate of overall survival up to 24 months. Full line shows the median value and shaded space shows the 95% CI.

**Figure 2** displays the Kaplan-Meier estimate of OS up to 24 months. At a median follow up of 13.3 months (95% CI: 11.8 to 15.7 months), a total of 32 (56.1%) deaths were observed. The median OS was 12.4 months (95% CI, 8.0 months to not reached). **Table 4** summarizes results of tumor response. Partial response was observed in 19 patients (33.3%), stable disease was observed in 16 patients (28.1%), while 22 patients (38.6%) experienced disease progression.

**Table 4:** Results of tumor response.

Tumor response	N = 57
Objective response, N (%)	
Complete response	0 (0.0%)
Partial response	19 (33.3%)
Stable disease, N (%)	16 (28.1%)
Progressive disease, N (%)	22 (38.6%)

Data are number (N) and percentage (%) of patients.

## 4 Discussion

Although sacituzumab govitecan has entered our daily practice based on positive results of the phase III ASCENT study, the safety and antitumor efficacy in a real-world population of patients are still under investigation. We reported the results of a multicenter, retrospective Italian analysis, demonstrating that sacituzumab govitecan is safe, active and effective in a real-world setting of patients with mTNBC.

In our analysis, the demographic characteristics of patients, the proportion of patients with TNBC at initial diagnosis, the median time from diagnosis to initiation of sacituzumab govitecan, the number of previous chemotherapy regimens and rates of previous use of PARP and PD-1/PD-L1 inhibitors, were similar to those reported in the ASCENT study (11). However, our study population had higher rates of lung (58% vs 46%), bone (51% vs. 20%) and lymph node (63% vs. 24%) metastases and a similar rates of liver metastases (39% vs. 42%) (11). Moreover, we included also 2 patients with ECOG ≥ 2 and 16 (28.1%) patients with brain metastases. Overall, these data suggest that patients treated according to clinical practice might have a worse prognosis compared to patients selected on the basis of the more rigorous predefined selection criteria of the regulatory ASCENT study, which included patients with stable brain metastases patients (not considered in the primary analysis of PFS), and excluded patients with ECOG PS >2 and with life expectancy <3 months).

In our study, sacituzumab govitecan demonstrated an acceptable safety profile, in line with that expected based on the pivotal trials. Hematological toxicities (anemia 67%, neutropenia 60%), alopecia (67%) and gastrointestinal toxicities (nausea 42%, diarrhea 39%) were the most common treatment-related AEs (any grade). Notably, the incidence of all gastrointestinal AEs was markedly lower than that reported for the sacituzumab govitecan group in the ASCENT (11) (nausea 57%, diarrhea 59%) and in the TROPiCS-02 (nausea 59%, diarrhea 62%) trials (15), as well as the incidence of grade 3-4 neutropenia observed in our analysis was lower than that of the ASCENT study (grade 3: 21% *vs* 34%; grade 4: 9% *vs* 17%) and of that listed in the label of the drug (7). Moreover, apart from 7.0% of patients with grade 3-4 elevation of transaminases, there were no other grade 3-4 hematological or gastrointestinal treatment-related AEs in more than one patient (1.7%), which is lower than that reported in the SmPC of the product (7).

The lower incidence of nausea and vomiting might be influenced by a better management of antiemetic prophylaxis adopted in clinical practice among our centers, in response to the high frequency of nausea/vomiting in the ASCENT study. In fact, more than two-thirds of patients (75.4%) started sacituzumab govitecan receiving a prophylactic treatment with corticosteroids, 5-HT3 and NK 1 receptor antagonists before the first cycle of therapy, while an additional 10.5% of patients needed it as a supportive measure because of experienced side-effects after the first cycle of therapy.

We also observed a lower incidence of any grade diarrhea and, in particular, of grade 3 diarrhea (only 2 patients) which could be correlated, at least in part, to the management with atropine in case of acute diarrhea onset during or shortly after infusion (4 patients, 7.0%) and with loperamide in case of delayed onset. We have also to consider that about a third of our patients (15 patients, 26,3%) were administered prophylactic atropine before the first cycle to prevent cholinergic syndrome. Moreover, subjective toxicity assessment in real-world cohorts may be affected by underreporting in medical records, as previously demonstrated (19, 20).

Regarding hematological toxicities, as above-reported, we observed a lower incidence of both grade 1-2 and grade ≥ 3 neutropenia, compared to that reported rates in published clinical trials. This finding may depend on several factors: (1) the increasing experience in the prevention or management of sacituzumab govitecan related toxicities in the clinical practice may have reduced the incidence of some severe AEs such as severe neutropenia; (2) in the real-world setting, dose reductions are more frequent, mostly due to less stringent protocols; this, in turn, may result in lowered toxicities, including hematological and non-hematological ones; (3) retrospective analyses could be limited by an under-reporting of the incidence and grading of AEs (20).

The routine testing of UGT1A1 polymorphisms is not recommended due to inconsistent data on cost-effectiveness, it was not checked for the majority of patients as per clinical practice. However, sixteen patients (28%) were tested for UGT1A1 polymorphisms; UGT1A1 variants was found in 4 patients, two of them experienced neutropenia (grade 3) and one patient experienced hypertransaminasemia (grade 4) and alopecia. Due to the small number of patients with UGT1A1 variants, no conclusion may be drawn regarding their predicted toxicity.

In the analysis of frequency of adverse effects, it should be also considered that the known labelled frequency distribution of adverse effects is mainly derived from the ASCENT study (11), which enrolled patient populations selected according to predetermined criteria, including the required recovery from previous toxicities and the requirement of adequate hematological, liver or renal function prior to enrolment, which suggests that the risk of development of adverse effects in a study conducted in a real-world setting in a less selected population may be higher than that observed in regulatory trials.

Globally our data, as well as the very low rate of permanent treatment discontinuations due to AEs (only 3 patients), confirm the safety of sacituzumab govitecan also in a real-world scenario, showing that most common toxicities (gastro-intestinal and hematological) can be effectively prevented and managed, optimizing patient care and treatment adherence (21).

Regarding the effectiveness of sacituzumab govitecan in this real-world cohort of patients, we found a median PFS of 4.9 months (95% CI, 3.7 to 7.1 months), at a median follow-up of 10.6 months, and a median OS of 12.4 months (95% CI, 8.0 months to not estimable), at a median follow-up of 13.3 months. Objective response rate was 33.3% (all responders had partial response), 28.1% of patients had stable disease, while 38.6% experienced disease progression.

In the contextualization of our results it is important to highlight that the primary efficacy analysis in the ASCENT trial (11) was conducted considering only patients without brain metastases. In this group (N=235), reported median PFS and OS were 5.6 months (95% CI, 4.1-5.8) and 12.1 months (95% CI: 10.7-14.0), respectively, with an ORR of 35%. However, when considering the entire sacituzumab govitecan arm of the study including patients with brain metastases (N=267), median PFS and OS were 4.8 months (95% CI, 4.1-5.8) and 11.8 months (95% CI, 10.5-13.8), respectively, in line with our reported real-world data (11).

Our study has some limitations that have to be highlighted: (1) the relatively small number of participants (57 evaluable patients), does not allow subgroups analyses and/or data adjustments based on some patients’ characteristics, such as the number of previous lines of chemotherapy, the presence of a previous diagnosis of mTNBC, age range and others; (2) in line with the observational nature of the study, the presence of known and unknown confounders cannot be excluded and adequately measured and controlled; (3) the conduction of the study only in few investigational sites in Italy could limit the generalization of results to other medical centers and to the entire Italian territory.

## 5 Conclusions

The results of this observational real-world analysis conducted in Italy provide further data supporting the role of sacituzumab govitecan in the management of mTNBC patients. In particular, our results suggest that the implementation of premedication and supportive measures reduces the risk of common adverse effects associated with the use of the drug in the clinical practice setting.

Finally, the effectiveness of sacituzumab govitecan demonstrated to be comparable to that observed in regulatory trials, despite the presence of worse prognostic factors in a non-selected real-world cohort of patients.

## Data availability statement

The raw data supporting the conclusions of this article are available at the following URL: https://zenodo.org/records/10728602. Further inquiries can be directed to the corresponding author/s.

## Ethics statement

The studies involving humans were approved by Reference IEC of each individual site. The studies were conducted in accordance with the local legislation and institutional requirements. The participants provided their written informed consent to participate in this study.

## Author contributions

RbC: Writing – review & editing. GB: Writing – review & editing. MP: Writing – review & editing. CM: Writing – review & editing. CD: Writing – review & editing. AR: Writing – review & editing. FP: Writing – review & editing. NS: Writing – review & editing. RdC: Writing – review & editing. TS: Writing – review & editing. PI: Writing – review & editing. FN: Writing – review & editing. AF: Writing – review & editing. DM: Writing – review & editing.
